# Increased isolation of nontuberculous mycobacteria among TB suspects in Northeastern, Tanzania: public health and diagnostic implications for control programmes

**DOI:** 10.1186/s13104-016-1928-3

**Published:** 2016-02-17

**Authors:** Abubakar S. Hoza, Sayoki G. M. Mfinanga, Arne C. Rodloff, Irmgard Moser, Brigitte König

**Affiliations:** Institute of Medical Microbiology and Epidemiology of Infectious Diseases, University of Leipzig, Liebigstrasse 21, 04103 Leipzig, Germany; Sokoine University of Agriculture, Morogoro, Tanzania; Muhimbili Centre, National Institute for Medical Research (NIMR), Dar es Salaam, Tanzania; Federal Research for Animal Health, Friedrich Loeffler Institut, Jena, Germany

**Keywords:** Nontuberculous mycobacteria, Peripheral diagnostic centres, Human immunodeficiency virus (HIV), Tanzania

## Abstract

**Background:**

Non-tuberculous mycobacteria (NTM) are increasingly reported worldwide associated with human disease. Defining the significance of NTM in settings with endemic tuberculosis (TB) requires the discrimination of NTM from TB in suspect patients. Correct and timely identification of NTM will impact both therapy and epidemiology of TB and TB-like diseases. The present study aimed at determining the frequency and diversity of NTM among TB suspects in northeastern Tanzania.

**Methods:**

A cross-sectional study was conducted between November 2012 through January 2013. Seven hundred and forty-four sputum samples were collected from 372 TB suspects. Detection was done by using phenotypic, GenoType^®^ Mycobacterium CM/AS kits, 16S rRNA and *hsp65* gene sequencing for identification of isolates not identified by Hain kits. Binary regression model was used to analyse the predictors of NTM detection.

**Results:**

The prevalence of NTM was 9.7 % of the mycobacterial isolates. Out of 36 patients with confirmed NTM infection, 12 were HIV infected with HIV being a significant predictor of NTM detection (*P* < 0.001). Co-infection with *Mycobacterium tuberculosis* (*M. tb*) was found in five patients. Twenty-eight NTM isolates were identified using GenoType^®^ Mycobacterium CM/AS and eight isolates could not be identified. Identified species included *M. gordonae* and *M. interjectum* 6 (16.7 %), *M. intracelullare* 4 (11.1 %), *M. avium* spp. and *M. fortuitum* 2 (5.5 %), *M. kansasii*, *M. lentiflavum*, *M. simiae*, *M. celatum*, *M. marinum* 1 (2.8 %) each. Of isolates not identified to subspecies level, we identified *M. kumamotonense* (2)*, M. intracellulare/kansasii, M. intermedium/triplex, M. acapulcensis/flavescens, M. stomatepiae, M. colombiense* and *M. terrae* complex (1) each using 16S rRNA sequencing. Additionally, *hsp*65 gene sequencing identified *M. kumamotonense*, *M. scrofulaceum*/*M. avium*, *M. avium*, *M. flavescens/novocastrense, M. kumamotonense*/*hiberniae*, *M. lentiflavum, M. colombiense/M. avium* and *M. kumamotonense/terrae/hiberniae* (1) each. Results of the 16S rRNA and *hsp*65 gene sequencing were concordant in three and discordant in five isolates not identified by GenoType^®^ Mycobacterium CM/AS.

**Conclusion:**

NTM infections may play a vital role in causing lung disease and impact management of TB in endemic settings. GenoType^®^ Mycobacterium CM/AS represents a useful tool to identify clinical NTM infections. However, 16S rRNA gene sequencing should be thought for confirmatory diagnosis of the clinical isolates. Due to the complexity and inconsistence of NTM identification, we recommend diagnosis of NTM infections be centralized by strengthening and setting up quality national and regional infrastructure.

## Background

Over the past decades, the prevalence of pulmonary nontuberculous mycobacteria (NTM) is increasingly reported worldwide. However, epidemiological and surveillance data of NTM infections are still limited [1]. Defining the epidemiology of NTM diseases in most resource poor settings like Tanzania is more challenging than its well documented relative *Mycobacterium tuberculosis* complex (MTC) [2].

Due to their ubiquitous presence in the environment, exposure to NTM is likely common, as they can colonize the respiratory tract without causing disease such that finding NTM in respiratory secretions does not necessarily have clinical implications in all patients [3–5]. Differentiating true NTM lung infection from contamination and/or colonization is difficult; hence, the presence of acid-fast bacilli (AFB) positive by microscopy of a respiratory sample or culture poses major diagnostic challenge. Increasingly, NTM are becoming recognized as true pathogens and important causes of human infections [6, 7].

Information on the role, contribution and burden of NTM in aetiology of TB–like syndromes is limited in many sub-Saharan African countries endemic to TB and HIV [8–10]. Lack of rapid and accurate methods to diagnose AFB positive pulmonary infections due to NTM results in misdiagnosis and mismanagement of pulmonary TB in such settings. Correct and timely identification of NTM is particularly urgent for both therapy and epidemiology, since infections with different mycobacterial species demand different management approaches [11, 12].

Tanzania is among the 22 high burden countries (HBCs) in the world with high prevalence of TB. On average, 61,500 new TB patients are notified annually [13]. Patients with AFB positive sputum or with chest radiographic findings presumptive of active TB, who do not respond to general antimicrobials, are generally assumed positive for TB. As a general guideline, patients are empirically treated with first line anti-TB drugs for 6 months.

Since several TB-like syndromes could also be due to NTM, inconclusive diagnosis of pulmonary TB would result in over-diagnosis of TB and hence miss–management of both TB and NTM infections.

Invention of DNA strip technology (line probe assays) based on reverse hybridization of PCR products to their complementary oligonucleotide probes has revolutionized the diagnosis of NTM. Commercial DNA strip assay kits GenoType^®^ Mycobacterium CM/AS (Hain Life science GmbH, Nehren, Germany); GenoType^®^ CM/AS for detection of common and additional NTM species have been widely and successfully used as rapid molecular tools in the diagnosis of NTM both in resource poor and developed countries. However, cross–reactivity of DNA probes between mycobacterial species has been reported, leading to incorrect diagnosis and treatment of patients [14, 15]. Use of molecular techniques targeting the 16S rRNA and *hsp*65 genes have been useful in diagnosis and speciation of NTM, including those which are dead or uncultivable [16].

The objective of the present study was therefore to determine the frequency and diversity of NTM among TB suspects in Northeastern Tanzania using conventional phenotypic methods, GenoType^®^ Mycobacterium CM/AS kits and 16S rRNA as well as *hsp65* gene sequencing.

Our findings suggest that NTM infections may play a vital role in causing lung disease and hence impacting management of TB in endemic settings like Tanzania. Therefore, the need to consider NTM in TB control programmes in such settings is urgent.

## Methods

### Study settings

The study was conducted in two peripheral diagnostic centres (PDCs) of Ngamiani and Makorora. The centres serve as primary catchment for TB diagnosis in Tanga municipal and two hospitals of Muheza Designated District Hospital (MDDH) and Bombo referral hospital. Tanga is among the top 10 regions with high TB notification in Tanzania with annual TB notification cases for the entire region being about 3852 cases, and the TB prevalence of 217/100,000 and 244/100,000 in urban and rural settings respectively as per national prevalence survey, 2012 [17].

### Study design

A cross-sectional study was carried out between November 2012 through January 2013. A total of 744 spot and morning sputum samples were collected from 372 TB suspected patients self–presenting at the PDCs and hospitals. All patients presenting to the diagnostic centre with any of the following symptoms were included: presences of symptoms suggestive of TB for a period of ≥2 weeks, night sweats, fatigue, unexpected loss of weight, and fever. Three sputum samples were collected from patients (one on the spot during the first visit, one early morning and another spot the following morning). Early morning sputa were stored at −20 °C and later transported to the Mycobacteriology laboratory at the University hospital Leipzig, Germany for culture and molecular identification.

### Smear microscopy and culture isolation

Direct smear microscopy using Ziehl Neelsen (ZN) or fluorescent stains were performed at the respective study sites by experienced laboratory technicians and the results recorded in accordance to the WHO/IUTLD and National Tuberculosis and Leprosy Programme (NTLP) guidelines [18, 19].

In Leipzig, all the sputum specimens were processed by the standard N-acetyl-L-cysteine (NALC)-NaOH method [20]. Briefly, 10 ml of 0.5 % N-acetyl-cysteine (NALC) solution was added to each sample, specimens were then incubated at room temperature on a shaker for 20 min, followed by addition of 30 ml of phosphate buffered saline (PBS) pH 6.8 for neutralization and subsequently centrifuged at 3000×*g* for 20 min. The sediments were resuspended in 1 ml PBS after discarding the supernatant. All the decontamination procedure followed the Deutsches Institut für Normung (DIN) recommendations for the detection of mycobacteria [21].

About 2–3 drops of resuspended specimens were inoculated on Lowenstein-Jensen (LJ), Gottsacker, and Coletsos slants (Artelt-ENCLIT GmbH, Germany) supplemented with antibiotics (Polymyxin B (200,000 IU/liter), Amphotericin B (10 mg/liter), Carbenicillin (50 mg/liter), and Trimethoprim (10 mg/liter) (PACT). Cultures were incubated for 8 weeks at 37 °C and read weekly.

Examination and reporting of smear microscopy was performed by fluorescent microscopy. Subsequently, 0.5 ml of each specimen was inoculated into BacT/Alert bottles supplemented with antibiotics PACT and incubated in an automated BacT/Alert 3D System (Biomérieux, Marcy l’Etoile, France) liquid culture system for 8 weeks. Culture not showing any growth after 8 weeks of incubation was considered negative. Positive BacT/Alert bottles were checked for purity by plating a drop from each positive bottle onto blood agar (BA) plate to rule out false positive results due to bacterial contamination and simultaneously ZN stain was performed to confirm presence of AFB positive results.

### Phenotypic identification

Slants containing pure cultures of AFB were assessed for growth rate and pigment accumulation on LJ, Gottsacker or Coletsos slants (at 30 °C, 37 °C and 45 °C). NTM isolates were grouped based on Runyon classification and results compared with those of molecular methods.

### DNA extraction

Mycobacterial DNA was extracted from heat–inactivated AFB isolates. Briefly, bacteria suspended in 500 µl sterile water or 1 ml directly from positive BacT/Alert bottles were inactivated at 80 °C for 20 min, then ultra-sonicated at 35 kHz and heated at 100 °C for 10 min each treatment and centrifuged at 16,100×*g* two times for 5 min. The supernatant was taken as template DNA. Genomic DNA of the H37Rv strain and sterile distilled water were used as positive and negative controls respectively for all molecular procedures.

### Identification of NTM by GenoType^®^ Mycobacterium CM/AS assay

All isolates identified as NTM based on their cultural characteristics and confirmed by ZN staining were subjected to further definitive identification using two commercial kits, the GenoType^®^ Mycobacterium CM for detection of common NTM. Isolates not identified by GenoType^®^ CM assay were further tested with the GenoType^®^ AS assay for additional NTM. All the procedures followed manufacturer’s instructions.

### DNA sequence analysis

All isolates not identified to the species level by the CM/AS assay were analyzed by 16S rRNA and *hsp*65 gene sequencing. For sequence analysis of the 16S rRNA gene was done according to [22] using the primers 285 and 264 for generating the PCR product (1037 bp) and primer 271 for sequencing. For *hsp*65 gene sequencing, PCR was done according to [23] using the primers 21M13TB11/M13TB12 generating a 441 bp fragment. For sequencing, the primer M13/pUC forward was used. The primers were purchased from Jena Bioscience, Jena, Germany. The PCR products were purified for sequencing using QIAquick PCR Purification Kit or alternatively QIAquick Gel Extraction Kit according to the manufacturer’s instructions. Sequencing was done by GATC Company (Konstanz, Germany). The raw data were analyzed at the FLI using the National Centre for Biotechnology Information (NCBI) BLAST software optimized for highly similar sequences (http://blast.ncbi.nlm.nih.gov). Strain identification was based on the BLAST hit with the highest scores combined with greatest sequence coverage and identity. Reference strains with the highest similarity score as present in the NCBI database were used as references for the similarity assessment.

### Data analysis

Demographic and clinical data were cleared and analysed by SPSS version 11.5 for Windows (SPSS Inc., Chicago, IL) software package. Binary regression model was used to analyse the predictors of NTM isolation. One-way analysis of variance (ANOVA) was used to determine trend analysis across ordered groups. *P* value <0.05 was considered statistically significant. GenoType^®^ Mycobacterium CM/AS assay results were interpreted based on manufacturers instruction.

## Ethical clearance

The protocol for this study was reviewed and approved by the Ethical Committee of the National Institute for Medical Research (NIMR), Dar es Salaam, Tanzania. Written informed consent was obtained from the patients or relatives of the patients, where the patients could not read and write.

## Results

A total of 744 sputum samples were collected from 372 TB suspects who self–presented at two PDCs and two hospitals in Tanga, northeastern, Tanzania. The proportion of males 196 (52.7 %) was higher than that of female 176 (47.3 %). The median age of the patients was 40 years (range 7–88 years).

### Demographic characteristics of patients and risk factors associated with NTM

Demographic data and risk factors associated with NTM infections are shown in (Tables 1, 2). The overall frequency of the patients with NTM in the study was 9.7 %; HIV positivity was found to be associated with NTM infection among the factors analysed, with a statistical significance of (OR 3.86, 95 % CI [1.79–8.3], *P* < 0.001). There was no association between NTM and other factors analysed.

**Table 1:** Demographic characteristics and laboratory findings of the patients with NTM infections stratified by site

Demographic and risk factors	Makorora HC n = 48 (%)	Ngamian HC n = 128 (%)	Bombo RH n = 62 (%)	Muheza DDH n = 134 (%)	Total n = 372
Gender					
Female	20 (41.7)	56 (43.7)	40 (64.5)	60 (44.8)	176
Male	28 (58.3)	72 (56.3)	22 (35.5)	74 (55.2)	196
Age: Mean (SD)	45.1 (18.9)	39.4 (17.6)	38.3 (15.6)	42.9 (16.9)	
Age groups (years)					
<20	1 (2.1)	8 (6.3)	6 (9.7)	6 (4.5)	21
20–40	13 (27.1)	69 (53.9)	23 (37.1)	51 (38.1)	156
40–60	22 (45.8)	29 (22.6)	27 (43.5)	57 (42.5)	135
>60	12 (25.0)	22 (17.2)	6 (9.7)	20 (14.9)	60
Residence					
Urban	39 (81.3)	97 (75.8)	43 (69.4)	28 (20.9)	207
Rural	9 (18.7)	31 (24.2)	19 (30.6)	106 (79.1)	165
Occupation					
Peasants	24 (50.0)	33 (25.8)	31(50.0)	68 (50.8)	156
Housewife	11 (22.9)	16 (12.5)	18 (29.0)	24 (17.9)	69
Business	5 (10.4)	24 (18.8)	5 (8.1)	11 (8.2)	45
Others	8 (16.7)	55 (42.9)	8 (12.9)	31 (23.1)	102
Prevalence of MTBC alone	5 (10.4)	29 (22.7)	15 (24.2)	36 (26.9)	85
Prevalence of NTM alone	5 (10.4)	14 (11.0)	1 (1.6)	11 (8.2)	31
Prevalence of MTB +NTM	0	3 (3.2)	1 (1.6)	1 (0.7)	5
Concurrent conditions					
HIV + ve	12 (25.0)	14 (10.9)	13 (30.0)	14 (10.4)	53
HIV − ve	7 (14.6)	87 (68.0)	1 (1.6)	19 (14.2)	114
Unknown	29 (60.4)	27 (21.1)	48 (77.4)	101 (75.4)	205
Previous TB					
Yes	9 (18.7)	33 (25.8)	1 (1.6)	16 (11.9)	59
No	39 (81.3)	95 (74.2)	61 (98.4)	118 (88.1)	313

*HC* health Centre, *MDDH* Muheza Designated District Hospital, *MTBC* *M. tuberculosis* complex, *NTM* nontuberculous mycobacterium

**Table 2:** Risk factors associated with NTM infection among suspected TB patients attending PDCs/hospitals in north-eastern, Tanzania from November 2012 to January 2013

	OR	95 % CI	*P value*
Gender	0.83	0.42–1.66	0.6
Age	1.02	0.52–2.04	0.95
Location	0.96	0.48–1.9	0.9
Occupation	0.95	0.43–2.1	0.8
HIV positive	3.86	1.79–8.3	0.001^*^
Previous TB	0.84	0.31–2.26	0.7

*OR* odd ratio, *CI* confidence interval

^*^ *P* value is statistically significant at *P* < 0.05

### Culture Results

Of the 372 patients, positive mycobacterial cultures were obtained in 121 (32.5 %) patients and among these 36 (9.7 %) patients harboured NTM, which are subject of this paper. From each patient three sputum samples were analysed for NTM growth. In total, 101 sputum samples were positive for NTM by culture. In this regard, 29 patients (80.6 %) were positive for NTM growth in all three samples and seven patients (19.4 %) were positive for NTM only in two out of the three samples. NTM without any other coinfections were detected in 21 (58.3 %) patients. NTM coinfection with HIV was found in 11 (30.6 %) patients, with MTBC in three (8.3 %) patients and NTM with both HIV and MTBC coinfections in one (2.8 %) patient. One patient was simultaneously positive for NTM plus *Nocardia* spp. (data not shown) (Table 3). None of the patients had mixed NTM infections.

**Table 3:** Comorbidity of NTM with either *M.tb* and or HIV among individuals with NTMs

Type of infection	No. of individuals (%)
NTM alone	21 (58.3 %)
HIV + NTM	11 (30.6 %)
NTM + *M.tb*	3 (8.3 %)
HIV + NTM + *M.tb*	1 (2.8 %)
Total	36

Of the 121 patients, 81 (66.9 %) patients had positive smear microscopy for AFB and 40 (33.1 %) patients were smear negative at the laboratory in Leipzig. On the other hand, of those 121 patients with positive culture results, 64 (52.9 %) patients had positive smear microscopy for AFB and 56 (46.3 %) patients had negative smear microscopy for AFB at local PDCs/hospitals, whereas only one (0.8 %) patient had smear positive AFB at local PDCs/hospitals but negative smear microscopy and culture in Leipzig.

### Phenotypic identification of the NTM

Based on their growth characteristics and pigment production, the isolates were grouped into different Runyon groups [24]. Eighteen isolates were classified as scotochromogenic (Runyon II), 13 as nonphotochromogenic (III), 4 as photochromogenic (Runyon I) and 1 as rapid growing mycobacteria (Runyon IV). The results were comparable to the molecular detection by Hain kit results (Table 4).

**Table 4:** Species distribution of the NTM isolated from PDCs/hospitals in northeastern Tanzania based on Runyon grouping and GenoType^®^ Mycobacterium CM/AS

Prevalence of NTM infection	Runyon group	n = 36 (%)	Assay
*M. gordonae*	II	6 (16.7)	GenoType CM
*M. interjectum*	II	6 (16.7)	GenoType CM
*M. intracellulare*	III	4 (11.1)	GenoType CM
*M. scrofulaceum*	II	3 (8.3)	GenoType CM
*M. avium* spp.	III	2 (5.5)	GenoType CM
*M. fortuitum*	III	2 (5.5)	GenoType CM
*M. kansasii*	I	1 (2.8)	GenoType AS
*M. lentiflavum*	II	1 (2.8)	GenoType AS
*M. simiae*	I	1 (2.8)	GenoType AS
*M. celatum*	III	1 (2.8)	GenoType AS
*M. marinum*	I	1 (2.8)	GenoType AS
NTM not identified		8 (22.2)	

### Distribution of mycobacterial isolates by site

Results of genotyping using GenoType^®^ MTBC for the MTC and GenoType^®^ Mycobacterium CM/AS for NTM isolates at each site showed that the prevalence of *M. tuberculosis* alone were 10.4, 22.7, 24.2 and 26.9 % at Makorora HC, Ngamiani HC, Bombo RH and MDDH respectively. The prevalence of NTM alone were 10.4, 11.0, 1.6 and 8.2 % at Makorora HC, Ngamiani HC, Bombo RH and MDDH respectively (Table 1).

### Identification of NTM isolates GenoType^®^ Mycobacterium CM/AS

Identification of NTM isolates to species level by GenoType^®^ Mycobacterium CM/AS kits was achieved in 28 (77.8 %) isolates with GenoType^®^ Mycobacterium CM identifying 23 (63.9 %) of the isolates and GenoType^®^ Mycobacterium AS identifying 5 (13.9 %) isolates. On the other hand, eight (22.2 %) out of 36 isolates could not be identified by either kit. *M. gordonae* and *M. interjectum* were the most frequently identified with 6 (16.7 %) isolates each, followed by *M. intracellulare* 4 (11.1 %), *M. scrofulaceum* 3 (8.3 %), *M. avium* spp. 2 (5.5 %), *M. fortuitum* 2 (5.5 %), *M. kansasii* 1 (2.8 %), *M. lentiflavum* 1 (2.8 %), *M. simiae* 1 (2.8 %), *M. celatum* 1 (2.8 %) and *M. marinum* 1 (2.8 %) (Table 4).

### 16S rRNA gene and *hsp*65 gene sequencing

Eight isolates not previously identified by the GenoType^®^ Mycobacterium CM/AS kits were analysed by 16S rRNA gene sequencing and additionally by *hsp*65 gene sequencing. Three of eight species yielded comparable results with both genes while five *hsp65* analyses gave results different from 16S rRNA gene sequencing.

16S rRNA gene sequencing for the eight isolates yielded *M. kumamotonense* (n = 2), *M. intracelullare/kansasii, M. intermedium/triplex*, *M. acapulcensis/favenscens*, *M. stomatepiae, M. colombiense* and *M. terrae* complex (n = 1). The results with the respective lengths of the sequenced DNA stretches are shown in (Table 5).

**Table 5:** NTM species identified based on 16S rRNA gene and *hsp*65 gene sequencing

Strain ID	16S rRNA gene sequencing	Identity/sequence length (bp)	*hsp*65 gene sequencing	Identity/sequence length (bp)
TZ095	*M. kumamotonense*	558/559	*M. kumamononense*	357/357
TZ141	*M. intracellulare*	922/928	*M. scrofulaceum*	343/359
	*M. kansasii*	924/928	*M. avium* complex	343/360
TZ145	*M.* *intermedium*	902/916	*M. avium* complex	300/315
	*M.* *triplex*	896/916		
TZ147	*M. acapulcensis*	918/918	*M. flavescens*	369/372
	*M. flavescens*	912/919	*M. novocastrense*	368/372
TZ149	*M. kumamotonense*	928/928	*M. kumamotonense*	356/357
			*M. hiberniae*	382/384
TZ217	*M. stomatepiae*	570/570	*M. lentiflavum*	362/372
TZ224	*M. colombiense*	928/928	*M. colombiense*	378/385
			*M. avium* complex	376/385
TZ294	*M. terrae* complex	923/931	*M. kumamotonense*	368/384
			*M. terrae*	364/384
			*M. hiberniae*	363/384

## Discussion

While the epidemiology of TB is well documented, the prevalence and epidemiology of NTM in Tanzania is largely unravelled [25]. NTM pulmonary infections are increasingly reported because of increased populations at-risk due to HIV infection, old age, other immunosuppressive conditions, increased awareness and improved diagnostic facilities especially in developed countries [26, 27].

In Tanzania, the existing supposition is that most individuals presenting with pulmonary symptoms reflecting mycobacterial diseases are infected with MTC. Chances that NTM are missed during diagnosis is to a great extent attributed to poor diagnostic capabilities for culture and identification of NTM, endemic nature of MTC, overburden by diseases like malaria and HIV. Furthermore, lack of awareness among public health personnel and lack of standardized or accepted criteria to properly define and report NTM have all resulted to less attention on the NTM infections.

In this study, the overall frequency of patients with NTM detected by Hain kits among pulmonary TB suspects population was 9.7 %. We identified *M. gordonae* and *M. interjectum* with 6 (16.7 %) isolates each accounting for about one-third of all NTM isolates, *M. intracelullare* 4 (11.1 %), *M. avium* spp. and *M. fortuitum* 2 (5.5 %), *M. kansasii*, *M. lentiflavum*, *M. simiae*, *M. celatum*, *M. marinum* 1 (2.8 %) isolate each. Additionally, eight isolates, which gave no signal with GenoType^®^ Mycobacterium CM/AS kits were identified by 16S rRNA gene sequencing. These isolates were assigned to the species *M. kumamotonense* (2), *M. intracellulare/kansaii* (1)*, M. intermedium/M. triplex* (1), *M. acapulcensis/M. flavescens* (1)*, M. stomatepiae* (1), *M. colombiense* (1) and *M. terrae* (1). Additionally, *hsp*65 gene sequencing was conducted if the 16S rRNA sequences were not discriminatory in order to confirm especially the ambiguous species assignments. However, only three of eight isolates both genes indicated the same species, for five isolates discordant results were achieved by sequencing both genes (Table 5). Sequencing of different genes used for bacterial species identification may yield inconsistent results. The problem is even more complicated with mycobacteria which are characterized by interspecies similarity clearly higher than in other bacteria [28, 29]. These results emphasise the difficulties of species identification for NTM. Therefore, it is of great importance to standardize the methods in order to generate comparable results between different laboratories, countries and continents.

*M. kumamotonense*, *M. acapulcensisM. novocastrense*, *M. stomatepiae*, and *M. hiberniae* determined by either 16S rRNA or *hsp*65 in this study (Table 5) have been reported elsewhere in the world. Some of which have been associated with human diseases and others isolated from the environment [30–33]. To the best of our understanding, these species have not previously reported in Tanzania.

Our findings are in agreement with findings from other studies in Tanzania and Africa which indicated increasing prevalence of NTM [9, 34, 35]. Whether there exist geographic variations in the diversity of NTM in Tanzania is not clear since this study focused only on patients residing in a more or less similar geographic location along the northeastern coast of Tanzania.

Understanding the distribution and clinical impact of NTM is of public health significance, as it addresses concerns of over diagnosis of tuberculosis and potential under treatment of NTM infections. As reported previously [35], there exist quite diverse species of NTM among humans, livestock and wildlife [36]; this therefore stresses need to investigate the distribution and clinical impact of different NTM species in Tanzania.

Analysis of the predictors of NTM infection in this study shows lack of association among gender, age, area of residence and occupation in patients diagnosed with NTM. However, need to study NTM infections in TB endemic settings like Tanzania through a larger cohort and evaluation of their impact on TB disease is particularly urgent. Though in the present study we did not perform drug susceptibility testing for the isolated NTM, such patients may erroneously regarded as having MDR isolates.

Immunocompromised individuals due to HIV/AIDS are at a high risk of NTM infections, with *M. intracellulare*, and *M. avium* complex (MAC) being frequently reported [37–39].

Findings from this study show that individuals with HIV positive status had a varying range of NTM infections and HIV was an important predictor of NTM detection (OR 3.86, 95 % CI [1.79–8.3], p < 0.001). The identified NTM included *M. scrofulaceum* (2), *M. avium* spp. (2), *M. gordonae* (2), *M. intracelullare* (1), *M. lentiflavum* (1), *M. celatum* (1) and *M. interjectum* (1). Two of the isolates from HIV positive cases, not identified by the Hain kits, were found to belong to *M. kumamotonense* and *M. intracellulare* by 16S rRNA sequencing.

MAC and rarely identified species of *M. lentiflavum* and *M. sherrisii* were reported among HIV positive patients with pulmonary disease in Zambia and Tanzania [9, 34, 40]. *M. sherrisii* which was found to be commonly associated with HIV individuals in northern Tanzania [34], was not detected in this patient population. *M. celatum* commonly isolated from human respiratory tract specimens is also known to be pathogenic to HIV patients and sometimes non–immunocompromised patients [41], whereas, *M. gordonae* a common contaminant of water supply, soil, casual resident in human sputum and gastric lavage specimens is rarely (e.g. AIDS) if ever implicated in disease processes [41].

NTM co-infections with *M. tuberculosis* disease are rarely diagnosed owing to overlapping clinical manifestations [10]. Our results show that five (1.3 %) individuals had both *M. tuberculosis* and NTM which included *M. scrofulaceum* (2), *M. interjectum* (1), *M. gordonae* (1) and one isolate not identified by Hain kits was identified as *M. kumamotonense* by 16S rRNA sequencing. Although it may be assumed that patients with such co-infections in many cases manifest symptoms mainly due to *M. tuberculosis,* the role of such co–infections underscore the need for further research to determine their contribution in the disease pathogenesis, severity and progression.

NTMs pose a major challenge for TB treatment programmes since such patients are managed mainly on the basis of smear microscopy which is not suitable to differentiate between MTC and NTM, but also major drawbacks lie on limited sensitivity and specificity of symptoms and radiology [8]. Culture on the other hand, is the gold standard but is time consuming, demands use of different types of media and longer incubation for optimization of growth. Since such elaborate culture algorithms are scarce outside the reference centres, NTMs warrant a special emphasis as possible cause of disease.

Although we cannot certainly conclude whether the isolated NTM merit to be classified as cause of infection/disease in each particular case, a mere presence of NTM in a particular case could make the decision on the diagnosis more complex. Need for public hygiene education is particularly high, since NTM are mostly found in the environment (water, soil). If people exert better hygiene management in their homes and food preparation, animal keeping, water boiling before drinking and so on, the risk of being infected would decrease, especially for persons at risk such as children, elderly, and immunocompromised individuals.

## Limitations

A number of factors could have limited our findings. Firstly, lack of preliminary laboratory data on the chronic patients presented limited us from determining whether they had been infected only by an NTM or they primarily had coinfection with *M. tuberculosis*. Secondly, lack of follow up limits the capacity to establish patients’ outcomes especially with NTM disease in many resource poor settings. Moreover, the true prevalence of NTM can only be assessed through a wider epidemiological study. These findings were communicated to the Medical Research Coordinating Committee where National Tuberculosis Control Program is represented. However, we are not sure if NTLP guided starting of appropriate treatment for NTM since there are no clear national guidelines for management of NTMs. This makes these findings very important as they may serve as baseline data for the NTLP to develop guidelines for management of NTMs.

In conclusion, our findings suggest that there is a diverse range of NTM infections, which may play a vital role in causing lung disease and impact the management of TB in TB-endemic settings leading to misdiagnosis and inappropriate treatment of MDR cases particularly the clinically ‘‘chronic cases’’. This highlights the need to consider NTM when treating patients with putative TB treatment failures. Moreover, fundamental information that meets ATS/IDSA diagnostic criteria for diagnosis of pulmonary NTM is needed to improve the understanding of NTM disease. There is an urgent need of formulating standardized criteria for defining and reporting NTM infections. We recommend that laboratory diagnosis of NTM infections be centralized by strengthening and setting up quality national and regional infrastructure.
